# Conventional fluoroscopy-guided versus zero-fluoroscopy catheter ablation of supraventricular tachycardias

**DOI:** 10.1186/s12872-022-02544-6

**Published:** 2022-03-13

**Authors:** Tine Prolič Kalinšek, Jernej Šorli, Matevž Jan, Matjaž Šinkovec, Bor Antolič, Luka Klemen, David Žižek, Andrej Pernat

**Affiliations:** 1 Cardiovascular Surgery Department, University Medical Centre Ljubljana, Zaloska cesta 7, 1000 Ljubljana, Slovenia; 2 Medical Faculty, University of Ljubljana, Vrazov trg 2, 1104 Ljubljana, Slovenia; 3 Cardiology Department, University Medical Centre Ljubljana, Zaloska cesta 7, 1000 Ljubljana, Slovenia

**Keywords:** Zero-fluoroscopy, Supraventricular tachycardia, Cryoablation, Three-dimensional electroanatomic mapping system, Paediatric population, Intracardiac echocardiography

## Abstract

**Purpose:**

The aim of this study was to evaluate the safety and efficacy of zero-fluoroscopy (ZF) catheter ablation (CA) for supraventricular tachycardias (SVT).

**Methods:**

584 consecutive patients referred to our institution for CA of SVT were analysed. Patients were categorised into two groups; zero-fluoroscopy (ZF) group and conventional fluoroscopy (CF) group. The ZF group was further divided into two subgroups (adults and paediatric). Patient characteristics, procedural information, and follow-up data were compared.

**Results:**

The ZF group had a higher proportion of paediatric patients (42.2% vs 0.0%; *p* < 0.001), resulting in a younger age (30.9 ± 20.3 years vs 52.7 ± 16.5 years; *p* < 0.001) and lower BMI (22.8 ± 5.7 kg/m^2^ vs 27.0 ± 5.4 kg/m^2^; *p* < 0.001). Procedure time was shorter in the ZF group (94.2 ± 50.4 min vs 104.0 ± 54.0 min; *p* = 0.002). There were no major complications and the rate of minor complications did not differ between groups (0.0% vs 0.4%; *p* = 0.304). Acute procedural success as well as the long-term success rate when only the index procedure was considered did not differ between groups (92.5% vs 95.4%; *p* = 0.155; 87.1% vs 89.2%; *p* = 0.422). When repeated procedures were included, the long-term success rate was higher in the ZF group (98.3% vs 93.5%; *p* = 0.004). The difference can be partially explained by the operators' preferences.

**Conclusion:**

The safety and efficacy of ZF procedures in adult and paediatric populations are comparable to that of CF procedures.

**Supplementary Information:**

The online version contains supplementary material available at 10.1186/s12872-022-02544-6.

## Introduction

CA with either radiofrequency (RF) energy or cryoenergy is a well-establish therapy in treating SVT in both paediatric and adult populations [1]. Traditionally, X-ray fluoroscopy is used during CA procedures, though the utilisation of ionising radiation carries non-negligible stochastic and deterministic risks to the health of both the patient and the professional staff. These effects are cumulative and behave in a linear no-threshold manner and, as such, are especially important in paediatric populations [2]. The importance of reducing ionising radiation exposure has been recognised by the American College of Cardiology, which recommends the ALARA (as low as reasonably achievable) principle in all interventional laboratories [3].

In recent years, advances in three-dimensional (3D) electroanatomical mapping (EAM) systems and their utilisation have enabled the near-zero and ZF approaches to be studied. In a recent multicentre randomised trial investigating the near-zero approach in right- and left-sided SVT ablation, reduction of fluoroscopy was achievable in all patients and resulted in an estimated 96% reduction in overall risk of cancer incidence and mortality. The study also estimated that the supplementary cost associated with the addition of the 3D EAM system is justified when increase in life expectancy and period of life without cancer are considered [4]. However, most data regarding the feasibility, safety, and efficacy of the ZF approach is available for right-sided SVT only [5–8] or for the left-sided SVT via retrograde transaortic approach [9, 10].

The aim of this study was to evaluate the safety and efficacy of ZF CA for the treatment of SVTs in adult and paediatric populations in comparison to the CF-based approach.

## Methods

### Patients

Our retrospective and comparative analysis included 584 consecutive patients who had an inducible and ablated SVT, including atrioventricular nodal reentrant tachycardia (AVNRT), atrioventricular reentrant tachycardia (AVRT), and atrial tachycardia (AT) between December 2014 and May 2019. Patients with a visible ventricular preexcitation and an ablation of the accessory pathway (AP) were also included regardless of tachycardia induction. Patients with atrial fibrillation and/or atrial flutter were excluded. In addition, patients with SVT and ventricular tachycardia or premature ventricular depolarization were excluded. Presence of cardiac implantable electronic device, structural heart disease, and/or presence of severe cardiac or thoracic anatomic anomaly were not exclusion criteria. Two groups of patients were analysed and the outcomes compared: the CF group, where X-ray fluoroscopy was used during CA procedures and the ZF group, where CA procedures were performed without the use of X-ray fluoroscopy. The ZF group was further divided into a ZF subgroup for adult patients and a ZF subgroup for paediatric patients. Paediatric patients aged ≤ 18 years were referred to ZF CA as a default. Adult patients were referred to either ZF or CF CA at the referring physician's discretion. Patient characteristics, procedural information, and follow-up data were collected and analysed. Written informed consent to undergo the CA was obtained from all patients, their parents, or legal guardians before the procedure. The ZF CA procedure protocol was approved by the national medical ethics committee. All patients underwent a pre-procedural clinical examination, routine blood biochemistry laboratory analysis, and AAD therapy was discontinued for a minimum of five half-lives of the active agent prior to the procedure. The discontinuation of amiodarone was left to the physician's discretion.

### Electrophysiological study

Patients over 14 years of age had procedures performed in conscious sedation, with the rest under general anaesthesia. Local anaesthesia was used for femoral vein access in all patients, which was obtained under ultrasound guidance at the operator's discretion. In the CF group, guidance and placement of the catheters was performed using fluoroscopic guidance. In addition, the 3D EAM system (Carto 3, Biosense Webster, Diamond Bar, CA, USA) and intracardiac echocardiography (ICE, AcuNav, Siemens Healthineers AG, Erlangen, GER) were used at the operator's discretion in the CF group. In the ZF group, only the 3D EAM system (Carto 3, Biosense Webster, Diamond Bar, CA, USA; EnSite NavX, EnSite Velocity, EnSite Precision, Abbott, St. Paul, MN, USA) was used for the guidance of catheters in right-sided SVTs. For left-sided SVTs, in addition to the 3D EAM, intracardiac echocardiography (ICE) (AcuNav, Siemens Healthineers AG, Erlangen, GER) was used for transseptal punctures in both groups, and was further used for navigation of the catheters at the operator's discretion.

After femoral vein access was obtained, a ten-polar steerable diagnostic catheter (Polaris X, Boston Scientific, Marlborough, MA, USA; ViaCath, Biotronik, Berlin, GER) was advanced from the femoral vein into the heart and inserted into the coronary sinus (CS). Next, a four-polar diagnostic catheter (MultiCath, Biotronik, Berlin, GER) was inserted into the heart and placed on the basal section of the right side of the interventricular septum. In the ZF group, the ten-polar diagnostic catheter was used to mark the location of His potential on the 3D EAM map and to construct a partial 3D model of the right atrium. In the CF group, an additional diagnostic catheter (MultiCath, Biotronik, Berlin, GER) was placed at the location of the His potential. Figures illustrating positioning of the catheters are available in the Additional file 1: Figures S1–S5 chapter Figures.

Atrio-ventricular (AV) and ventriculo-atrial (VA) conduction testing followed. Atrial pacing was performed with the ten-polar diagnostic catheter in the CS and ventricular stimulation with the four-polar catheter at the basal interventricular septum. Atrial programmed stimulation and fast atrial stimulation were performed with the aim of tachycardia induction. If induction of tachycardia was not achieved or conduction over the AP was not detected, the protocol was repeated with an isoprenaline challenge. In cases of clear ventricular preexcitation, the induction of tachycardia was left to the physician's discretion. Standard diagnostic maneuvers were employed as needed to determine the type of induced tachycardia.

### Transseptal puncture

**Fig. 1:**
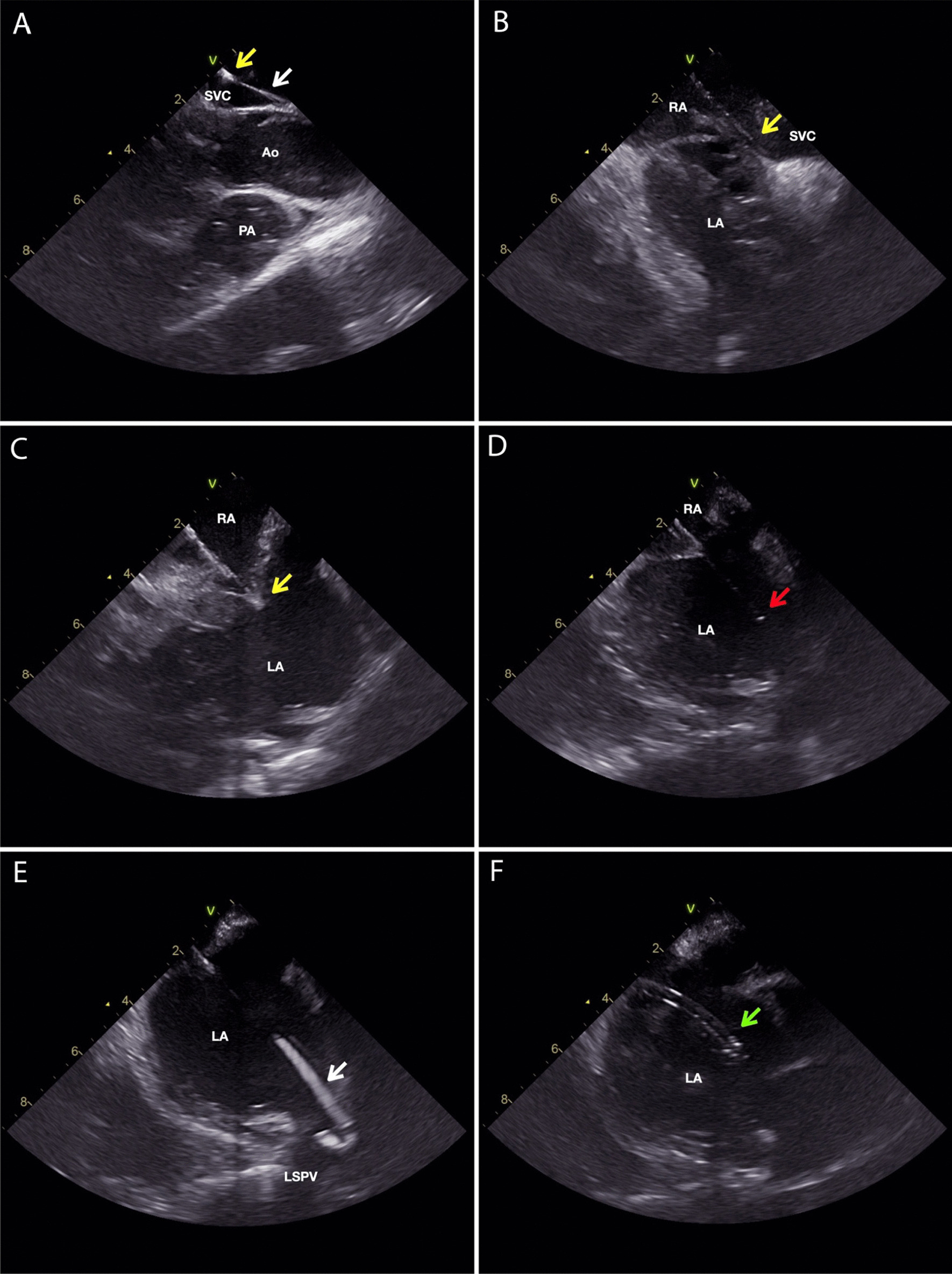
ICE images of the zero fluoroscopy transseptal puncture guided by ICE only. Guide wire is marked with a white arrow, the tip of the dilator is marked with a yellow arrow, the tip of the transseptal needle is marked with a red arrow, and the tip of the long sheath is marked with a green arrow. **A** The guide wire is advanced through the SVC and the long sheath with a dilator fully inserted is advanced over the guide wire into the SVC. The ICE probe is located in the SVC just above the RA and SVC junction. **B** The guide wire is removed and the transseptal needle is not fully inserted into the long sheath with 1–2 cm of the needle outside the sheath. The long sheath / dilator / transseptal needle assembly is pulled down from the SVC to the interatrial septum. **C** The tip of the dilator is positioned on the oval fossa. At this moment, a tenting of the interatrial septum can be observed. **D** the needle is pushed fully into the long sheath. Transseptal puncture is performed. **E** the transseptal needle is withdrawn and the guidewire is advanced through the dilator into the LSPV. **F** the guide wire and the dilator are withdrawn with the tip of the long sheath in the LA. Ao—aorta; LA—left atrium; LSPV—left superior pulmonary vein; PA—pulmonary artery; RA—right atrium; SVC—superior vena cava

ICE was used to visualise the position of the guide wire and later the position and the course of the long sheath/dilator/transseptal needle assembly during the pull-down maneuver from the superior vena cava. The aortic root, pulmonary veins, and the interatrial septum visualised with ICE were used as endovascular landmarks. Posteroinferior part of the oval fossa opposite to the posterior aspect of the left pulmonary vein antrum was the target for the transseptal puncture (Fig. 1). ICE was also used to confirm the presence, orientation, and exact location of the transseptal needle tip, the dilator tip, the long sheath tip, and the guide wire in the left atrial cavity during different stages of transseptal puncture.

### Cryoablation of AVNRT

Cryoablation was used in the ZF group in AVNRT cases at the physician’s discretion. A 4-mm or 6-mm tip cryocatheter (Freezor and Freezor Xtra, Medtronic, Minneapolis, MN, USA) was used. Cryomapping (− 30 °C) was first performed during ongoing tachycardia or during programmed atrial stimulation with manifest conduction over the slow pathway. If the tachycardia terminated during cryomapping or the conduction over the slow pathway was terminated, the cryomapping was then switched to cryoablation (− 80 °C), usually for 240 s. An additional lesion was applied in close proximity to the successful one. If the tachycardia or slow pathway conduction were terminated mechanically during cryoablation, the location was tagged on the 3D EAM system and additional lesions were applied at the spot of mechanical termination when possible.

### Procedural endpoints

The procedural endpoint for AVNRT was noninduction, with and without the isoprenaline challenge. Presence of slow pathway conduction with up to one “echo” beat was allowed. The procedural endpoint for AVRT ablation was the elimination of AV and VA conduction across the AP. Additionally, noninduction of tachycardia was always tested with or without an isoprenaline challenge. For AT, the procedural endpoint was termination with ablation and noninduction of tachycardia with or without an isoprenaline challenge.

### Procedural complications

Major complications were defined as events which were directly related to the CA procedure and required an intervention, prolonged hospital stay, and/or had a negative influence on the patient's long-term health. Minor complications were defined as a transient high-degree atrioventricular block that resolved during the procedure, pericardial effusion without a hemodynamic compromise requiring no intervention, and other adverse events that would not be qualified as major complications but were still directly related to the CA procedure. Predischarge transthoracic ultrasound examination of the pericardial space was performed in all patients that underwent transseptal puncture.

### Follow-up

All patients received post-procedural instructions for further actions in case of recurrence. During subsequent outpatient appointments, patients underwent clinical examinations and had a 12-lead ECG recorded. If the patient had signs and symptoms of recurrence of tachycardia, further diagnostic tests were performed, including 24-h holter monitoring, monitoring with wearable event recorders, and/or repeated EP study. Recurrences were confirmed and noted during the EP study. If the patient declined repeated EP study, the tachycardia recorded on 12-lead ECG or 24-h holter was noted as a recurrence.

### Statistical analysis

Descriptive data of continuous variables are presented as mean ± standard deviation or as median and interquartile range (25–75%). Categorical variables are presented as numbers with percentages. Differences between groups were evaluated by an independent Student’s t-test for normally distributed continuous variables and the Mann–Whiteny U test for non-normally distributed continuous variables, while χ^2^ was used for categorical variables. Categorical variables with fewer than two values were compared with Fisher’s exact test. A one-way ANOVA test was used for the analysis of the learning curve. Time to first recurrence of tachycardia was plotted using the Kaplan–Meier product and compared by the log-rank test. All statistical analyses were performed using SPSS (IBM, Armonk, NY, USA) statistical software version 25. A *p* value of < 0.05 was considered statistically significant.

## Results

### Patients

A total of 280 patients were categorised into the CF group, and 294 into the ZF group. Of these, 170 were in the ZF adult subgroup and 124 in the ZF paediatric subgroup. The baseline characteristics are shown in Table 1. The CF and ZF groups differed in mean age (52.7 ± 16.5 years vs 30.9 ± 20.3 years, *p* < 0.001) and BMI (27.0 ± 5.4 kg/m^2^ vs 22.8 ± 5.7 kg/m^2^, *p* < 0.001). In the subgroup analysis, the differences of mean age and BMI were present only between the CF group and the ZF paediatric subgroup. There was only one patient younger than 5 years, aged 4 years at the time of the procedure. There were no patients with a body weight below 15 kg; the lightest patient weighed 18 kg. The CF and ZF groups differed in the type of tachycardia: the ZF group had a lower proportion of patients with AVNRT (53.1% vs 63.0%, *p* < 0.016) and a higher proportion of patients with AVRT (34.4% vs 23.8%, *p* = 0.006); these differences were present in the ZF paediatric subgroup but not in the ZF adult subgroup.

**Table 1:** Comparison of baseline characteristics between the CF and ZF groups and the ZF subgroups (ZF subgroup adults—consisting of adult patients only; ZF subgroup paediatric—consisting of paediatric patients only)

	CF group	ZF group	ZF subgroup adults	ZF subgroup paediatric
Number of patients (n)	280	294	170	124
Underage patients (n, %)	0/280 (0.0)	124/294 (42.2)*	0/170 (0.0)	124/124 (100)*
Female (n, %)	121/280 (43.2)	145/294 (49.3)	76/170 (44.7)	69/124 (55.6)*
Age (years)	52.7 ± 16.5	30.9 ± 20.3*	44.1 ± 17.1	12.8 ± 3.61*
BMI (kg/m^2^)	27.0 ± 5.4	22.8 ± 5.7*	26.0 ± 5.4	19.4 ± 3.63*
AVNRT (n, %)	177/280 (63.2)	156/294 (53.1)*	96/170 (56.5)	60/124 (48.4)*
AVRT (n, %)	66/280 (23.6)	101/294 (34.4)	48/170 (28.2)	53/124 (42.7)
AT (n, %)	37/280 (13.2)	37/294 (12.6)	26/170 (15.3)	11/124 (8.9)
Multiple arrhythmias (n, %)	12/280 (4.2)	8/294 (2.7)	8/170 (4.7)	0/124 (0.0)*
AAD (n, %)	157/280 (56.1)	61/294 (20.7)*	41/170 (24.1)*	20/124 (16.1)*
amiodarone (n, %)	7/280 (2.5)	2/294 (0.7)	2/170 (1.2)	0/124 (0.0)
nonamiodarone (n, %)	152/280 (54.3)	58/294 (19.7)*	38/170 (22.4)*	20/124 (16.1)*
*p* value^a^				
	ZF group		ZF subgroup adults	ZF subgroup paediatric
Underage patients (n, %)	< 0.001		NA	< 0.001
Female (n, %)	0.143		0.757	0.021
Age (years)	< 0.001		0.758	< 0.001
BMI (kg/m^2^)	< 0.001		0.052	< 0.001
AVNRT (n, %)	0.014		0.156	0.005
AVRT (n, %)	0.004		0.270	< 0.001
AT (n, %)	0.822		0.538	0.213
Multiple arrhythmias (n, %)	0.307		0.834	< 0.001
AAD (n, %)	< 0.001		< 0.001	< 0.001
amiodarone (n, %)	0.078		0.328	0.075
nonamiodarone (n, %)	< 0.001		< 0.001	< 0.001

AAD—antiarrhythmic drug; AP—accessory pathway; AT—atrial tachycardia; AVNRT—atrioventricular nodal reentry tachycardia; AVRT—atrioventricular tachycardia; CF—conventional fluoroscopy-guided; ZF—zero-fluoroscopy

^*^A statistically significant difference (*p* value < 0.05)

^a^The ZF group, ZF subgroup adults, and ZF subgroup paediatric were all compared with the CF group

There were also fewer patients on AAD therapy in the ZF group at the time of the index procedure (20.7% vs 56.1%, *p* < 0.001). There were no crossovers in either of the groups.

### Procedural data

**Fig. 2:**
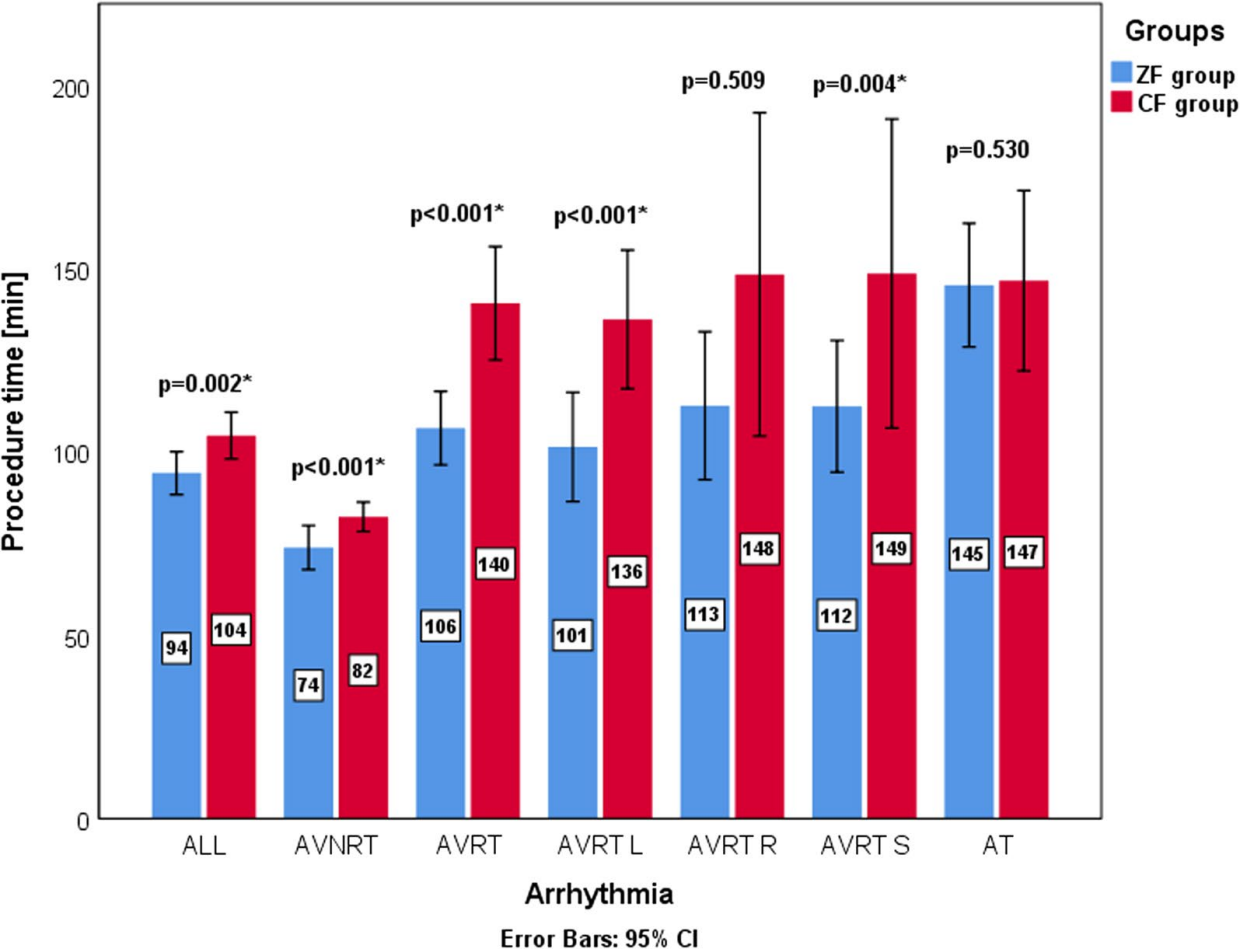
A clustered bar graph for procedure time of different arrhythmias. The blue bars represent mean procedure time for the ZF group. The red bars represent mean procedure time for the CF group. The whisker bars represent 95% confidence intervals. The mean procedure time of each group is presented by the numbers superimposed on the bars. An asterisk next to a *p* value signifies a statistically significant difference (*p *< 0.05)

**Fig. 3:**
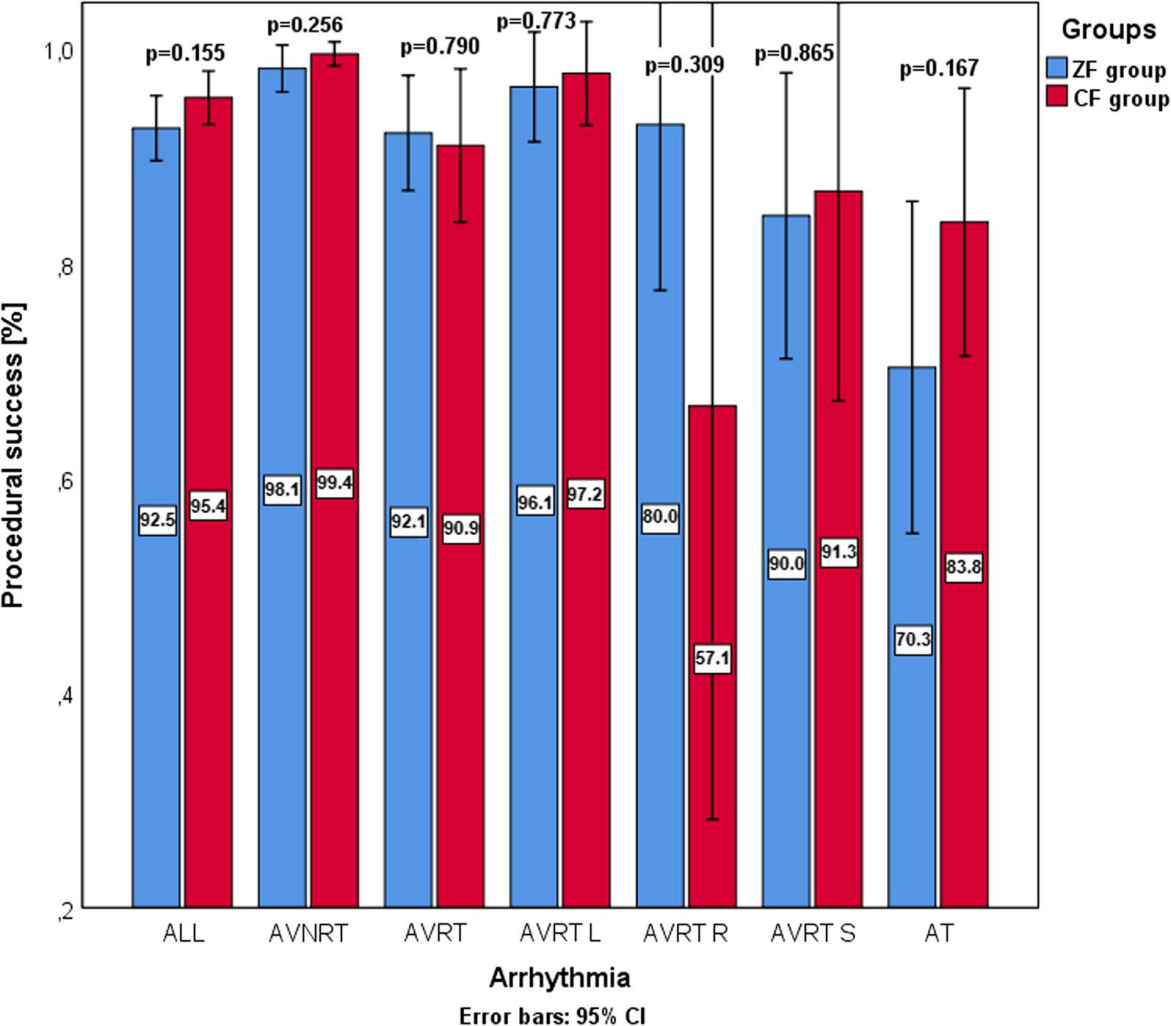
A clustered bar graph for the procedural success of different arrhythmias. The blue bars represent the percentages of arrhythmia-free survival in the ZF group. The red bars represent the percentages of arrhythmia-free survival in the CF group. The whisker bars represent 95% confidence intervals. There were no statistically significant differences between the groups

There was a statistically significant difference between utilisation of the 3D EAM system between the CF and ZF group and ZF subgroups. The difference was also present when looking at each type of 3D EAM system (Carto 3, Biosense Webster, Diamond Bar, CA, USA vs EnSite, Abbott, St. Paul, MN, USA), with the exception being comparison between utilisation of Carto 3 system in CF group and ZF adult subgroup (5.4% vs 2.9%; *p* = 0.345). Additionally, there were significantly more cryoablation and significantly fewer RF ablation procedures in the ZF group (cryoablation: 44 vs 0; RF ablation: 250 vs 280, *p* < 0.001). When looking at RF ablation procedures, the median number of lesions (8 (4–17) vs 7 (4–13), *p* = 0.015) and ablation time were significantly higher in the ZF group (382 ± 379 s vs 233 ± 242 s, *p* < 0.001). These differences were present in the ZF adult subgroup and not in the ZF paediatric subgroup. Procedure time was shorter in the ZF group (94.2 ± 50.4 min vs 104.0 ± 54.0 min; *p* = 0.002). Differences of procedure time for different arrhythmias can be seen in Fig. 2. This difference was present in both of the ZF subgroups. In the CF group, the average fluoroscopy time was 13.9 ± 11.0 min and the average dose area product (DAP) was 606 ± 1003 mGym^2^. There were no major complications in either group. One patient in the CF group who was treated for left atrial AT and had transseptal puncture developed pericardial effusion that resolved without an additional intervention. The two groups did not differ in overall procedural success rate (ZF vs CF; 92.5% vs 95.4%, *p* = 0.155). In Fig. 3, the procedural success for each arrhythmia can be found. In the subgroup analysis, patients in the ZF adult subgroup who underwent RF ablation for AVNRT had a statistically lower success rate compared to the CF group (95.8% vs 99.4%, *p* = 0.040). Detailed procedural information is available in the Additional file 1: Table S1.

### Learning curve analysis of the ZF approach

**Fig. 4:**
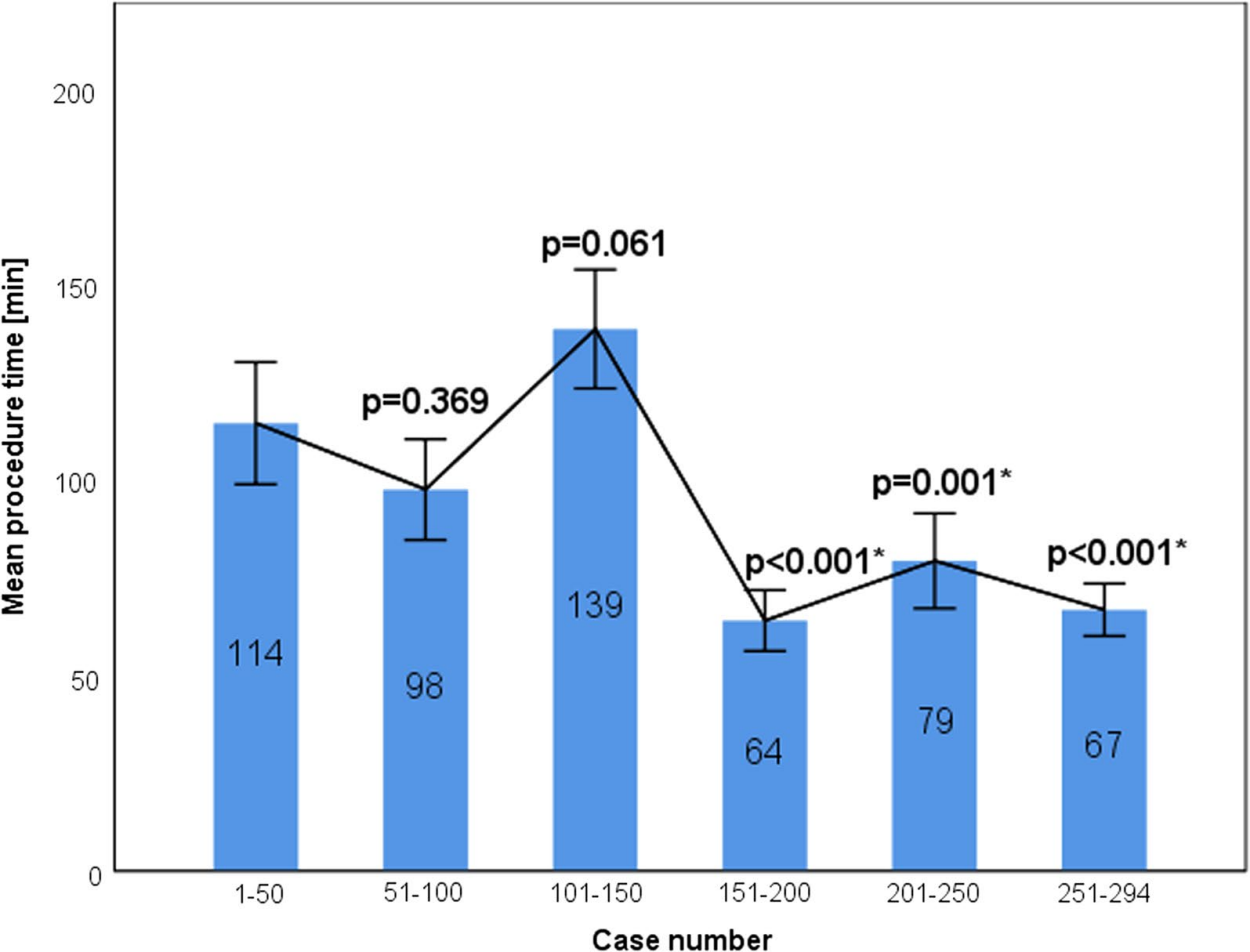
Graph displaying boxplots of mean procedure time per year and an interpolating line. A statistically significant difference was reached after 150 cases. Statistically significant procedure times are marked with an asterisk next to the *p* value. Mean procedure times are noted in each bar

An analysis of mean procedure time showed a statistically significant difference after the first 150 cases that remained significant in subsequent procedures. As shown in Fig. 4, a downward slope of the interpolating line can be observed with a significant drop after the first 150 cases. The results from the statistical analysis of the learning curve can be found in the Additional file 1: Table S2.

### Follow-up

#### Analysis of outcomes after index procedure

**Fig. 5:**
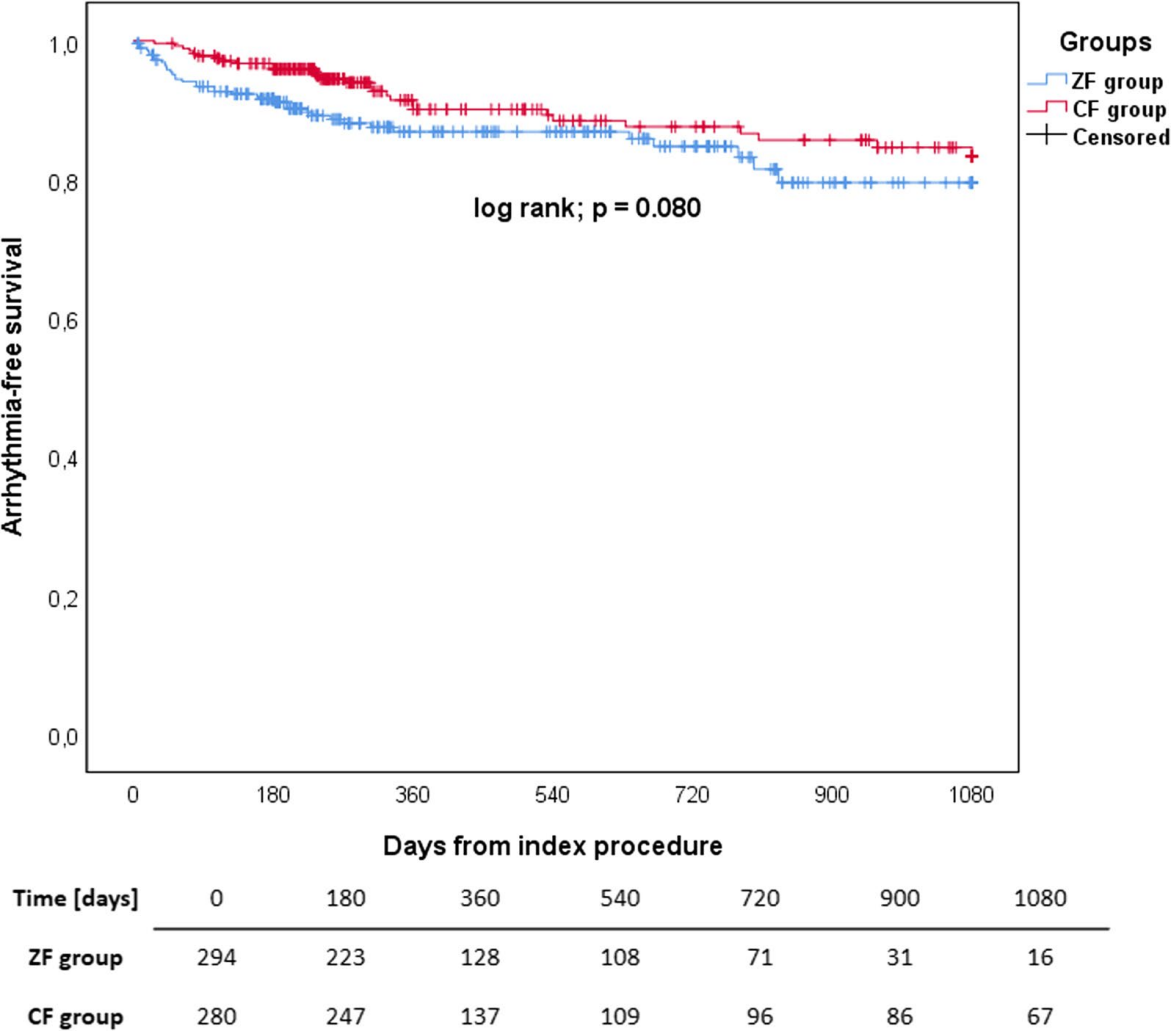
A Kaplan–Meier curve with the at-risk table of arrhythmia-free survival of the ZF and CF group after the index ablation. There were no statistically significant differences between the groups (ZF vs CF; 87.1% vs 89.2%; log rank *p* = 0.903)

The mean follow-up for both groups was 378 ± 306 days. There were significantly fewer patients on AAD therapy in the ZF group (14.3% vs 36.1%, *p* < 0.001). The groups did not differ in arrhythmia-free survival rates (Fig. 5). Detailed information is presented in the Additional file 1: Table S3.

#### Analysis of outcomes with repeated procedures included

**Fig. 6:**
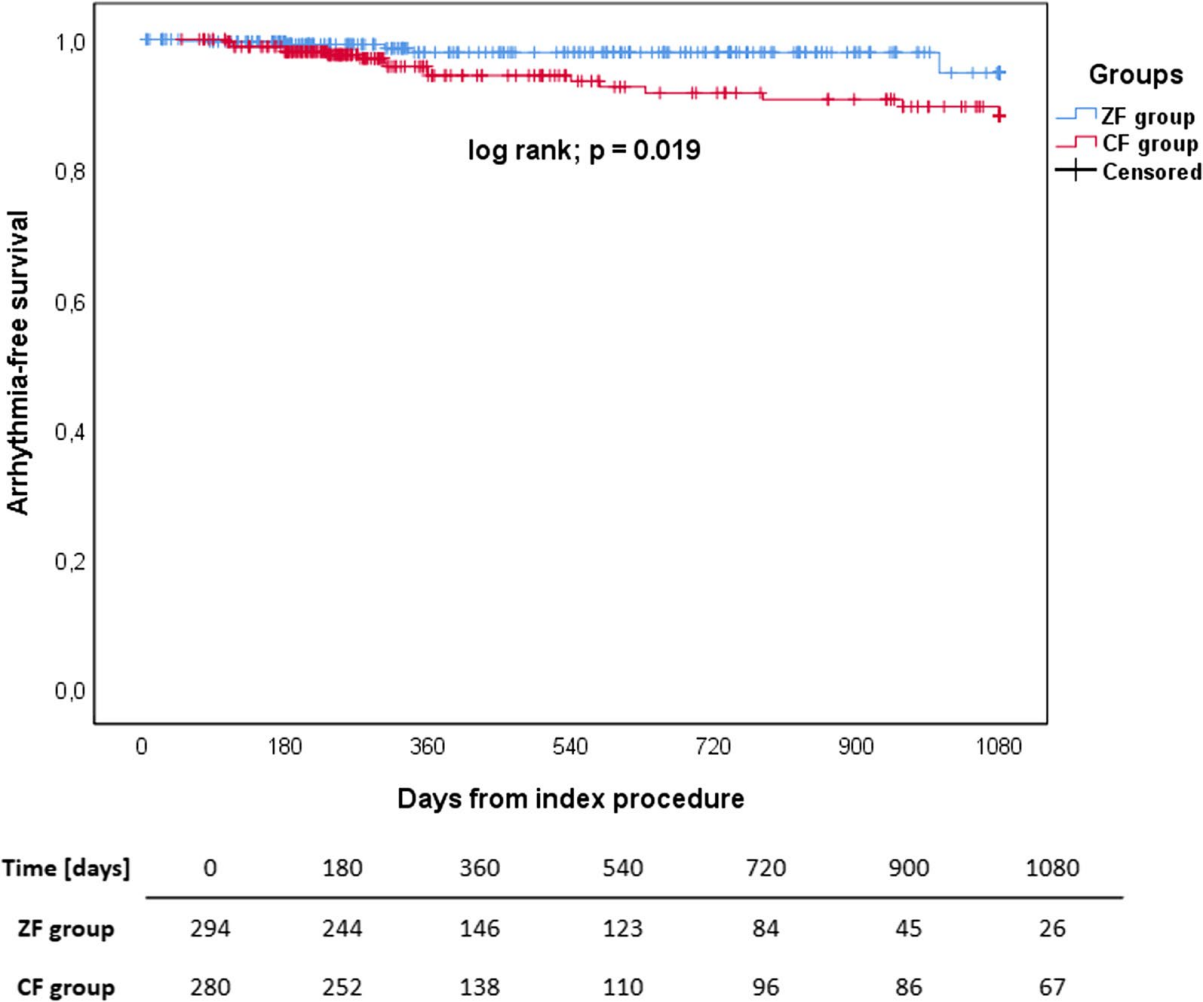
A Kaplan–Meier curve with the at-risk table of arrhythmia-free survival of the ZF and CF group after all procedures. There was a statistically significant difference between the groups (ZF vs CF; 98.3% vs 93.5%; log rank *p* < 0.001)

The mean follow-up for both groups was 424 ± 338 days. In the ZF group, there were fewer patients on AAD therapy (12.9% vs 36.1%; *p* < 0.001). The overall success rate was higher in the ZF group (98.3% vs 93.5%, *p* = 0.004). A Kaplan–Meier curve of arrhythmia-free survival is shown in Fig. 6. This difference was present in the ZF adult subgroup only. The overall success rate was also higher in patients without AAD therapy (98.4% vs 90.8%, *p* < 0.001), a difference present in all ZF subgroups. More specifically, there was a higher AVRT-free survival rate (99.0% vs 90.9%, *p* = 0.011) in the ZF group. This difference was present only in the ZF adult subgroup. There were also more procedures per patient in the ZF group (1.13 ± 0.356 vs 1.05 ± 0.241, *p* = 0.002). Detailed information is presented in the Additional file 1: Table S4.

## Discussion

The results of our analysis show comparable outcomes between the ZF and CF approaches for the treatment of SVTs with regards to efficacy and safety. The rate of complications was low among both groups, with no major procedural complications occurring.

The differences in the mean age and mean BMI of patients between groups in our study can be explained by the inclusion of paediatric patients in the ZF group only, an explanation confirmed by the ZF subgroup analysis. The rationale behind such a decision is that the stochastic and deterministic effects of ionising radiation are especially harmful to paediatric patients. Due to their developing bodies and long-life expectancy post-exposure, they are more susceptible to developing cancer [11]. As such, we referred them to ZF CA as a first choice. Interestingly, some differences in the use of AAD therapy before and after the CA between both groups were discovered. These differences cannot be explained by the inclusion of paediatric patients as evidenced by the analysis of the ZF paediatric and adult subgroups. It can be hypothesised that, since the same physicians both referred patients for CA procedures and performed post-procedural follow-up, there was some degree of individual physician preference for the use of AADs.

Regarding the procedural data, we found a statistically significant difference in procedural duration, with the mean duration being lower in the ZF group (94.2 ± 50.4 min vs 104.0 ± 54.0 min, *p* = 0.002). Our findings are in line with previous studies investigating the ZF approach in various SVTs, which reported the mean procedural duration to range from 50 to 129 min [6, 10–15]. Perhaps in contrast with the available literature, we found the procedural duration to be lower in almost all types of arrhythmias and locations of the substrate except in right-sided APs. We hypothesise that the features of the 3D EAM system not present in the CF approach are the cause of the shorter procedure times. Firstly, the ability to annotate the location of previously effective or ineffective ablation lesions facilitates the decision-making process during the CA procedure in almost all cases. Secondly, the distance between the His-bundle annotation and ablation catheter can be constantly monitored from two different views, which can be especially helpful in AVNRT, as well as for mid-septal and para-Hisian location of APs. Finally, local activation time mapping can identify the location of APs or the origin of AT both more rapidly and accurately. Additionally, our analysis of the learning curve showed that procedure time drops significantly after the first year of experience. Features of the 3D EAM system can perhaps explain the higher median number of RF lesions in the ZF group (8 (4–17) vs 7 (4–13), *p* = 0.015). We hypothesise that the ability to localise the successful ablation site with the 3D EAM system can prompt the operator to add additional adjacent bonus lesions. Without the use of the 3D EAM system, the exact location characterisation can be challenging. However, the difference was only present in the ZF adult subgroup (11 (6–20) vs 7 (4–13), *p* < 0.001). We suggest that the smaller hearts of paediatric patients as well as the potential for important heart structures such as the AV node and His bundle to be closer to the successful ablation site may prevent the operator from performing additional ablations despite the advantages of using the 3D EAM system. There were no statistically significant differences between the two groups regarding the overall procedural success rates. In the subgroup analysis, the procedural success rate in patients who underwent RF ablation for AVNRT was lower in the ZF adult subgroup (95.8% vs 99.4%, *p* = 0.040). However, recurrence rates during follow-up after index procedure in those groups were similar.

A relatively lower procedural success rate for AT in the ZF group (70.3% vs 83.8%, *p* = 0.167) and a relatively higher procedural success rate for right-sided APs in the ZF adult subgroup (100% vs 57.1%, *p* = 0.091) did approach but not reach statistically significant difference. The number of patients with AT and right-sided APs was also too low to be representative and useful for any further statistical analysis. In addition, we found that both groups had no major complications and no statistically significant difference in minor complication rate (CF vs ZF; 0.4% vs 0.0%, *p* = 0.304). Importantly, the reduction of radiation dose was substantial (CF; DIA: 13.9 ± 11.0 min; DAP: 606 ± 1003 mGym^2^; *p* < 0.001). These results are in line with the findings from previously published studies, adding to the body of evidence on the feasibility and safety of ZF SVT ablation [6, 10, 12–18].

Regarding long-term outcomes, there were no statistically significant differences between groups when only the data from the index procedure was analysed. The overall success rate did not differ between groups (ZF vs CF; 87.1% vs 89.2%, *p* = 0.422). This is also in line with previously published studies that reported no differences between the ZF and CF approach [10, 12, 13, 16]. When the data on repeated procedures was included, the overall long-term outcomes differed between the groups, being significantly higher in the ZF group (98.3% vs 93.5%; *p* = 0.004). This was also true when only patients with AVRT were analysed (99.0% vs 90.8%; *p* = 0.010). In the subgroup analysis of procedures for AVRT, this difference was notable only in the ZF adult subgroup (100% vs 90.8%, *p* = 0.031). These differences can be attributed to the higher number of procedures per patient in the ZF group (1.13 ± 0.356 vs 1.05 ± 0.234; *p* = 0.001) improving the overall success rate with included repeat procedures. As with the explanation for differences in the use of AADs, these differences in outcomes cannot be explained by the inclusion of paediatric patients, as evidenced by the analysis of the ZF paediatric and adult groups. It can again be hypothesised that in cases where tachycardia recurred, there was some individual physician preference for referring patients for repeat procedures versus the use of AADs.

An important finding that we would like to address is that CA procedures of AVNRT performed with cryoenergy had successful long-term outcomes in both analyses (100% after index procedure; 100% after repeated procedures were included). This is perhaps in contrast with the available published data. In a meta-analysis of cryoablation versus RF ablation for AVNRT, the long-term recurrence rate was significantly higher (9.7% vs 3.8%; *p* = 0.003) in patients ablated with cryoenergy [19]. We can speculate that the high success rate of ZF cryoablation in our study can, at least partially, be explained with the use of the 3D EAM system, which enables the operator to mark the location of a possible mechanical termination of the targeted slow pathway by the cryocatheter [20]. With this approach, the site of the mechanical block can be accurately ablated, in contrast to the CF approach where the exact location of the mechanical block can be more difficult to localise. An additional factor adding to the high success rate may be the utilisation of cryomapping during the ongoing AVNRT with tachycardia termination during ongoing cryoablation serving as an endpoint. This technique was previously published by Eryazici et al. and demonstrated a high long-term success rate [21].

Lastly, there are some limitations of the ZF approach when only a 3D EAM system is used for guidance. One such instance is that of transseptal punctures. To overcome the limitations of the 3D EAM system, the use of ICE for transseptal puncture is mandatory in our institution [22].

With ICE guidance, the operator can successfully identify true endovascular borders and anatomical structures as well as their relation to the transseptal needle. The usefulness of ICE, however, goes beyond guiding the transseptal puncture; with visual inspection, the presence of possible masses in the left atrium, on the long sheath, and on the catheter inserted into the left atrial cavity can be identified and further appropriate actions can be taken, possibly preventing certain potential procedural complications. It also offers precise guidance in manipulating catheters in the heart, including supervising the stability of the ablation catheter during lesion formation. Importantly, the pericardial space can be readily visually inspected for early detection of pericardial effusion.

## Limitations

Our study has several limitations. Due to the nature of retrospective studies, our results may have been influenced by selection bias. Three factors need to be addressed here: first, the inclusion of paediatric patients exclusively in the ZF group, which might have influenced the study results. However, several studies showed similar procedural outcomes compared to adult patients [23, 24]. Furthermore, additional analysis of our ZF subgroups comparing baseline characteristics and procedural outcomes did not show significant differences between adult and pediatric patients. Second, cryoenergy was used only in the ZF group. This may also have an impact on the procedural success rate. Also, the learning curve related to the ZF procedures might have impacted procedural parameters and outcomes in comparison to the CF procedures. Third, there were differences in utilisation of 3D EAM systems between the groups and subgroups, as seen in the Additional file 1: Table S1. However, recent study showed no difference in procedural and outcome data between different 3D EAM systems [15]. Finally, all data was collected at a single centre; as such, the results may not be directly applicable to experiences in other populations.

## Conclusions

Our study demonstrated that the safety and efficacy of ZF CA procedures for right and left-sided SVTs is comparable to that of the CF CA approach. To further assess the non-inferiority of the ZF approach compared to the CF approach, a randomised trial comparing both methods is needed.

## Supplementary Information

**Additional file 1.** Figures S1–S5 chapter Figures.

## Data Availability

We will share the data on reasonable request to the corresponding author.
