# Supplementary material for: Conventional fluoroscopy-guided versus zero-fluoroscopy catheter ablation of supraventricular tachycardias
Source: BMC Cardiovasc Disord. 2022 Mar 13;22:98. doi: 10.1186/s12872-022-02544-6 (PMC8919640; doi:10.1186/s12872-022-02544-6)
Supplement: Supplementary file 1 — Additional file 1. Figures S1–S5 chapter Figures. [file 12872_2022_2544_MOESM1_ESM.docx]

**Supplemental data**

**Table of Contents**

**Tables 1.02**

Table S12

Table S24

Table S35

Table S48

**Figures 2.010**

Figure S110

Figure S211

Figure S312

Figure S413

Figure S514

**Tables 1.0**

Table S1: Comparison of procedural data between groups. The ZF group was further divided into two subgroups (ZF subgroup adults – consisting of adult patients only; ZF subgroup paediatric – consisting of paediatric patients only).

|  | **CF group** | **ZF group** | **ZF subgroup adults** | **ZF subgroup paediatric** |
| --- | --- | --- | --- | --- |
| **Procedural data** | | | | |
| RF (n, %) | 280/280 (100) | 250/294 (85.0)^*^ | 139/170 (81.8)^*^ | 111/124 (89.5)^*^ |
| Cryoablation (n, %) | 0/280 (100) | 44/294 (15.0) | 31/170 (18.2) | 13/124 (10.5) |
| No. of RF lesions (n) | 7 (4 – 13) | 8 (4 – 17)^*^ | 11 (6 – 20)^*^ | 7 (4 – 12) |
| No. of cryoablation lesions (n) | 0 (0 – 0) | 3 (3 – 4) | 3 (3 – 5) | 3 (2 – 3) |
| RF ablation time (s) | 233 ± 242 | 382 ± 379^*^ | 441 ± 430^*^ | 303 ± 281^*^ |
| Cryoablation time (s) | 0 ± 0 | 837 ± 398 | 871 ± 347 | 744 ± 524 |
| Transseptal puncture (n, %) | 51/280 (18.2) | 73/294 (24.8) | 40/170 (23.5) | 33/124 (26.6) |
| Fluoroscopy time (min) | 13.9 ± 11.0 | 0 ± 0 | 0 ± 0 | 0 ± 0 |
| DAP (mGym^2^) | 606 ± 1003 | 0 ± 0 | 0 ± 0 | 0 ± 0 |
| 3D EAM system used (n, %) | 15/280 (5.4) | 294/294 (100)^*^ | 170/170 (100)^*^ | 124/124 (100)^*^ |
| Carto 3 | 15/280 (5.4) | 6/294 (2.0)^*^ | 5/170 (2.9) | 1/124 (0.8)^*^ |
| EnSite | 0/280 (0.0) | 288/294 (98.0)^*^ | 165/170 (97.1)^*^ | 123/124 (99.2)^*^ |
| **Procedure time** | | | | |
| Overall procedure time (min) | 104.0 ± 54.0 | 94.2 ± 50.4^*^ | 95.9 ± 52.2^*^ | 91.9 ± 48.0^*^ |
| AVNRT (min) | 82.3 ± 26.9 | 73.9 ± 37.6^*^ | 73.8 ± 34.4^*^ | 74.1 ± 42.5^*^ |
| AVRT (min) | 140.5 ± 62.4 | 106.4 ± 50.5^*^ | 109.7 ± 53.7^*^ | 103.5 ± 47.9^*^ |
| *left-sided AP (min)* | 130.8 ± 61.3 | 97.0 ± 50.1^*^ | 96.5 ± 53.8^*^ | 97.4 ± 47.6^*^ |
| *right-sided (min)* | 138.6 ± 61.9 | 121.0 ± 31.6 | 117.0 ± 22.5 | 125.0 ± 41.2^*^ |
| *septal AP (min)* | 156.8 ± 63.7 | 114.5 ± 53.6^*^ | 123.7 ± 57.1 | 106.2 ± 50.0^*^ |
| AT (min) | 146.6 ± 73.8 | 145.4 ± 50.5 | 150.8 ± 55.2 | 132.7 ± 36.2 |
| **Procedural success** | | | | |
| Overall procedural success (n, %) | 267/280 (95.4) | 272/294 (92.5) | 156/170 (91.8) | 116/124 (93.5) |
| AVNRT (n, %) | 176/177 (99.4) | 153/156 (98.1) | 93/96 (96.9) | 60/60 (100) |
| *RF AVNRT (n, %)* | 176/177 (99.4) | 124/127 (97.6) | 69/72 (95.8)^*^ | 55/55 (100) |
| *cryoablation AVNRT (n, %)* | 0/0 (0.0) | 29/29 (100) | 24/24 (100) | 5/5 (100) |
| AVRT (n, %) | 60/66 (90.9) | 93/101 (92.1) | 45/48 (93.8) | 48/53 (90.6) |
| *left-sided AP (n, %)* | 35/36 (97.2) | 49/51 (96.1) | 22/24 (91.7) | 27/27 (100) |
| *right-sided AP (n, %)* | 4/7 (57.1) | 8/10 (80.0) | 5/5 (100) | 3/5 (60.0) |
| *septal AP (n, %)* | 21/23 (91.3) | 36/40 (90.0) | 18/19 (94.7) | 18/21 (85.7) |
| AT (n, %) | 31/37 (83.8) | 26/37 (70.3) | 18/26 (69.2) | 8/11 (72.7) |
| **Complications** | | | | |
| Major complications (n, %) | 0/280 (0.0) | 0/294 (0.0) | 0/170 (0.0) | 0/124 (0.0) |
| Minor complications (n, %) | 1/280 (0.4) | 0/294 (0.0) | 0/170 (0.0) | 0/124 (0.0) |

| ***p* value ^a^** | | | |
| --- | --- | --- | --- |
|  | ZF group | ZF subgroup adults | ZF subgroup paediatric |
| RF | <0.001 | <0.001 | <0.001 |
| Cryoablation | <0.001 | <0.001 | <0.001 |
| No. of RF lesions | 0.015 | <0.001 | 0.591 |
| No. of cryoablation lesions | <0.001 | <0.001 | <0.001 |
| RF ablation time | <0.001 | <0.001 | 0.002 |
| Cryoablation time | <0.001 | <0.001 | <0.001 |
| Transseptal puncture | 0.054 | 0.174 | 0.055 |
| Fluoroscopy time | <0.001 | <0.001 | <0.001 |
| DAP | <0.001 | <0.001 | <0.001 |
| 3D EAM system used | <0.001 | <0.001 | <0.001 |
| Carto 3 | 0.034 | 0.345 | 0.044 |
| EnSite | <0.001 | <0.001 | <0.001 |
| Overall procedure time | 0.002 | 0.017 | 0.006 |
| AVNRT | <0.001 | 0.002 | 0.001 |
| AVRT | <0.001 | 0.002 | <0.001 |
| *left-sided AP* | 0.001 | 0.002 | 0.006 |
| *right-sided AP* | 0.509 | 0.422 | 0.680 |
| *septal AP* | 0.004 | 0.058 | 0.003 |
| AT | 0.530 | 0.418 | 1.000 |
| Overall procedural success | 0.155 | 0.120 | 0.450 |
| AVNRT | 0.256 | 0.093 | 0.560 |
| *RF AVNRT* | 0.175 | 0.040 | 0.576 |
| *cryoablation AVNRT* | <0.001 | <0.001 | <0.001 |
| AVRT | 0.790 | 0.579 | 0.949 |
| *left-sided AP* | 0.773 | 0.333 | 0.383 |
| *right-sided AP* | 0.309 | 0.091 | 0.921 |
| *septal AP* | 0.865 | 0.667 | 0.560 |
| AT | 0.167 | 0.171 | 0.409 |
| Major complications | NA | NA | NA |
| Minor complications | 0.304 | 0.435 | 0.504 |

^a^ The ZF group, ZF subgroup adults, and ZF subgroup paediatric were all compared with the CF group.

^*^ a statistically significant difference (*p* value < 0.05)

3D EAM – three-dimensional electroanatomic model; AAD – antiarrhythmic drug; AP – accessory pathway; AT – atrial tachycardia; AVNRT – atrioventricular nodal reentry tachycardia; AVRT – atrioventricular tachycardia; CF – conventional fluoroscopy-guided; DAP – dose area product; RF – radiofrequency; ZF – zero-fluoroscopy.

Table S2: An analysis of the learning curve.

|  |  | **Mean difference** | ***p value*** |
| --- | --- | --- | --- |
| 1-50 | 51-100 | 16.9 | 0.369 |
|  | 101-150 | -24.1 | 0.061 |
|  | 151-200 | 50.4^*^ | <0.001 |
|  | 201-250 | 35.1^*^ | 0.001 |
|  | 251-294 | 47.6^*^ | <0.001 |
| 51-100 | 1-50 | -16.9 | 0.369 |
|  | 101-150 | -41.0^*^ | <0.001 |
|  | 151-200 | 33.5^*^ | 0.002 |
|  | 201-250 | 18.1 | 0.297 |
|  | 251-294 | 30.7^*^ | 0.010 |
| 101-150 | 1-50 | 24.1 | 0.061 |
|  | 51-100 | 41.0^*^ | <0.001 |
|  | 151-200 | 74.5^*^ | <0.001 |
|  | 201-250 | 59.2^*^ | <0.001 |
|  | 251-294 | 71.7^*^ | <0.001 |
| 151-200 | 1-50 | -50.4^*^ | <0.001 |
|  | 51-100 | -33.5^*^ | 0.002 |
|  | 101-150 | -74.5^*^ | <0.001 |
|  | 201-250 | -15.3 | 0.489 |
|  | 251-294 | -2.8 | 1.000 |
| 201-250 | 1-50 | -35.1^*^ | 0.001 |
|  | 51-100 | -18.1 | 0.297 |
|  | 101-150 | -59.2^*^ | <0.001 |
|  | 151-200 | 15.3 | 0.489 |
|  | 251-294 | 12.5 | 0.732 |
| 251-294 | 1-50 | -47.6^*^ | <0.001 |
|  | 51-100 | -30.7^*^ | 0.010 |
|  | 101-150 | -71.7^*^ | >0.001 |
|  | 151-200 | 2.8 | 1.000 |
|  | 201-250 | -12.5 | 0.732 |

A one-way ANOVA test was used to compared the groups. ^*^ – a statistically significant difference.

Table S3: Comparison of follow-up data after index procedure between groups. The ZF group was further divided into two subgroups (ZF subgroup adults – consisting of adult patients only; ZF subgroup paediatric – consisting of paediatric patients only).

|  | **CF group** | **ZF group** | **ZF subgroup adults** | **ZF subgroup paediatric** |
| --- | --- | --- | --- | --- |
| **Follow-up data** | | | | |
| Duration of follow-up (days) | 362 ± 266 | 390 ± 333 | 314 ± 293^*^ | 482 ± 357^*^ |
| AAD (n, %) | 74/205 (36.1) | 42/294 (14.3)^*^ | 36/170 (21.2)^*^ | 6/124 (4.8)^*^ |
| amiodarone (n, %) | 2/205 (1.0) | 3/294 (1.0) | 3/170 (1.8) | 0/124 (0.0) |
| nonamiodarone (n, %) | 73/205 (35.6) | 41/294 (13.9)^*^ | 34/170 (20.0)^*^ | 7/124 (5.6)^*^ |
| **Success rate** | | | | |
| Overall success rate (n, %) | 249/279 (89.2) | 256/294 (87.1) | 150/170 (88.2) | 106/124 (85.5) |
| without AADs (n, %) | 115/131 (87.8) | 223/252 (88.5) | 120/134 (89.6) | 103/118 (87.3) |
| AVNRT (n, %) | 166/177 (93.8) | 143/156 (91.7) | 90/96 (93.8) | 53/60 (88.3) |
| *RF AVNRT (n, %)* | 166/177 (93.8) | 114/127 (89.8) | 66/72 (91.7) | 48/55 (87.3) |
| *cryoablation AVNRT (n, %)* | 0/0 (0.0) | 29/29 (100) | 24/24 (100) | 5/5 (100) |
| AVRT (n, %) | 54/66 (81.8) | 87/101 (86.1) | 42/48 (87.5) | 45/53 (84.9) |
| *left AP (n, %)* | 32/36 (88.9) | 48/51 (94.1) | 22/24 (91.7) | 26/27 (96.3) |
| *right AP (n, %)* | 4/7 (57.1) | 7/10 (70.0) | 4/5 (80.0) | 3/5 (60.0) |
| *septal AP (n, %)* | 18/23 (78.3) | 32/40 (80.0) | 16/19 (84.2) | 16/21 (76.2) |
| AT (n, %) | 29/36 (80.6) | 26/37 (70.3) | 18/26 (69.2) | 8/11 (72.7) |

| ***p* value ^a^** | | | |
| --- | --- | --- | --- |
|  | ZF group | ZF subgroup adults | ZF subgroup paediatric |
| Duration of follow-up (days) | 0.344 | <0.001 | 0.037 |
| AAD (n, %) | <0.001 | 0.002 | <0.001 |
| amiodarone (n, %) | 0.961 | 0.507 | 0.270 |
| nonamiodarone (n, %) | <0.001 | <0.001 | <0.001 |
| Overall success rate (n, %) | 0.422 | 0.741 | 0.282 |
| without AADs (n, %) | 0.839 | 0.650 | 0.905 |
| AVNRT (n, %) | 0.456 | 0.991 | 0.168 |
| *RF AVNRT (n, %)* | 0.200 | 0.548 | 0.115 |
| *cryoablation AVNRT (n, %)* | <0.001 | <0.001 | <0.001 |
| AVRT (n, %) | 0.452 | 0.411 | 0.654 |
| *left AP (n, %)* | 0.377 | 0.725 | 0.282 |
| *right AP (n, %)* | 0.585 | 0.408 | 0.921 |
| *septal AP (n, %)* | 0.870 | 0.625 | 0.870 |
| AT (n, %) | 0.308 | 0.304 | 0.579 |

^a^ The ZF group, ZF subgroup adults, and ZF subgroup paediatric were all compared with the CF group.

^*^ a statistically significant difference (*p* value < 0.05)

AAD – antiarrhythmic drug; AP – accessory pathway; AT – atrial tachycardia; AVNRT – atrioventricular nodal reentry tachycardia; AVRT – atrioventricular tachycardia; CF – conventional fluoroscopy-guided; RF – radiofrequency; ZF – zero-fluoroscopy.

Table S4: Comparison of follow-up data after all procedures are included. The ZF group was further divided into two subgroups (ZF subgroup adults – consisting of adult patients only; ZF subgroup paediatric – consisting of paediatric patients only).

|  | **CF group** | **ZF group** | **ZF subgroup adults** | **ZF subgroup paediatric** |
| --- | --- | --- | --- | --- |
| **Follow-up data** | | | | |
| Duration of follow-up (days) | 369 ± 267 | 467 ± 380 | 369 ±330 | 588 ± 402^*^ |
| AAD (n, %) | 74/205 (36.1) | 38/294 (12.9)^*^ | 35/170 (20.6)^*^ | 3/124 (2.4)^*^ |
| amiodarone (n, %) | 1/205 (0.5) | 2/294 (0.7) | 2/168 (1.2) | 0/124 (0.0) |
| nonamiodarone (n, %) | 73/205 (35.6) | 35/294 (11.9)^*^ | 32/170 (18.8)^*^ | 3/124 (2.4)^*^ |
| **Success rate** | | | | |
| Overall success rate (n, %) | 261/279 (93.5) | 289/294 (98.3)^*^ | 168/170 (98.8)^*^ | 121/124 (97.6) |
| without AADs | 119/131 (90.8) | 252/256 (98.4)^*^ | 134/135 (99.3)^*^ | 118/121 (97.5)^*^ |
| AVNRT (n, %) | 167/177 (94.4) | 152/156 (97.4) | 94/96 (97.9) | 58/60 (96.7) |
| *RF AVNRT (n, %)* | 167/177 (94.4) | 123/127 (96.9) | 70/72 (97.2) | 53/55 (96.4) |
| *cryoablation AVNRT (n, %)* | 0/0 (0.0) | 29/29 (100) | 24/24 (100) | 5/5 (100) |
| AVRT (n, %) | 59/65 (90.8) | 100/101 (99.0)^*^ | 48/48 (100)^*^ | 52/53 (98.1) |
| *left AVRT (n, %)* | 35/36 (97.2) | 51/51 (100) | 24/24 (100) | 27/24 (100) |
| *right AVRT (n, %)* | 5/7 (71.4) | 10/10 (100) | 5/5 (100) | 5/5 (100) |
| *septal AVRT (n, %)* | 19/22 (86.4) | 39/40 (97.5) | 19/19 (100) | 20/21 (95.2) |
| AT (n, %) | 35/37 (94.6) | 37/37 (100) | 26/26 (100) | 11/11 (100) |
| Number of procedures per patient | 1.05 ± 0.241 | 1.13 ± 0.356^*^ | 1.13 ± 0.370^*^ | 1.13 ± 0.337^*^ |

| ***p* value ^a^** | | | |
| --- | --- | --- | --- |
|  | ZF group | ZF subgroup adults | ZF subgroup paediatric |
| Duration of follow-up (days) | 0.167 | 0.079 | <0.001 |
| AAD (n, %) | <0.001 | <0.001 | <0.001 |
| amiodarone (n, %) | 0.784 | 0.456 | 0.436 |
| nonamiodarone (n, %) | <0.001 | <0.001 | <0.001 |
| Overall success rate (n, %) | 0.004 | 0.009 | 0.093 |
| without AADs | <0.001 | 0.001 | 0.025 |
| AVNRT (n, %) | 0.161 | 0.170 | 0.479 |
| *RF AVNRT (n, %)* | 0.305 | 0.337 | 0.556 |
| *Cryoablation AVNRT (n, %)* | <0.001 | <0.001 | <0.001 |
| AVRT (n, %) | 0.010 | 0.031 | 0.093 |
| *left AVRT (n, %)* | 0.231 | 0.410 | 0.383 |
| *right AVRT (n, %)* | 0.072 | 0.190 | 0.190 |
| *septal AVRT (n, %)* | 0.088 | 0.095 | 0.317 |
| AT (n, %) | 0.152 | 0.228 | 0.431 |
| Number of procedures per patient | 0.002 | 0.008 | 0.006 |

^a^ The ZF group, ZF subgroup adults, and ZF subgroup paediatric were all compared with the CF group.

^*^ a statistically significant difference (*p* value < 0.05)

AAD – antiarrhythmic drug; AP – accessory pathway; AT – atrial tachycardia; AVNRT – atrioventricular nodal reentry tachycardia; AVRT – atrioventricular tachycardia; CF – conventional fluoroscopy-guided; RF – radiofrequency; ZF – zero-fluoroscopy.

**Figures 2.0**

**
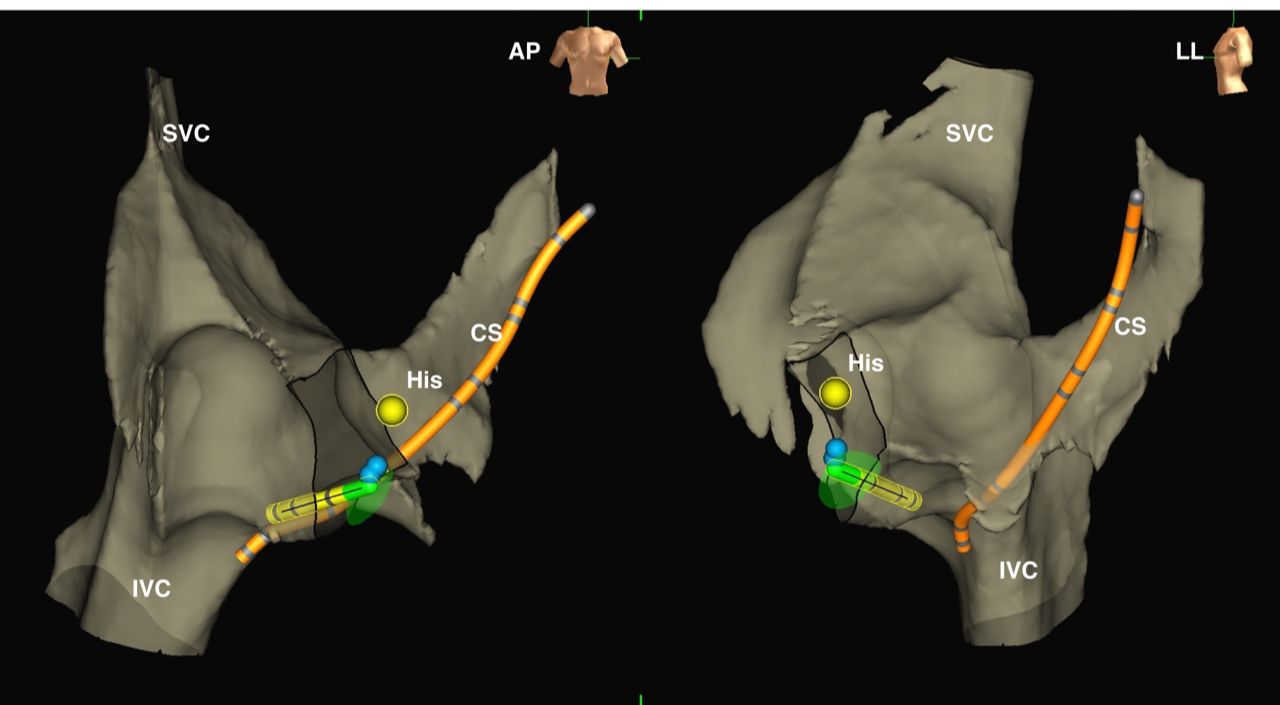
**Figure S1: Partial anatomical 3D map of the right atrium in a patient with AVNRT that was treated with cryoablation. The left panel displays the anteroposterior view and the right panel displays the left lateral view. Blue dots mark the successful cryoablation site at the presumed location of the slow pathway in the midseptal region. Also, a shadow of the cryoablation catheter tip is shown at the successful ablation site. AP – anteroposterior; CS – coronary sinus; His – His bundle; IVC – inferior vena cava; LL – left lateral; SVC – superior vena cava.

**
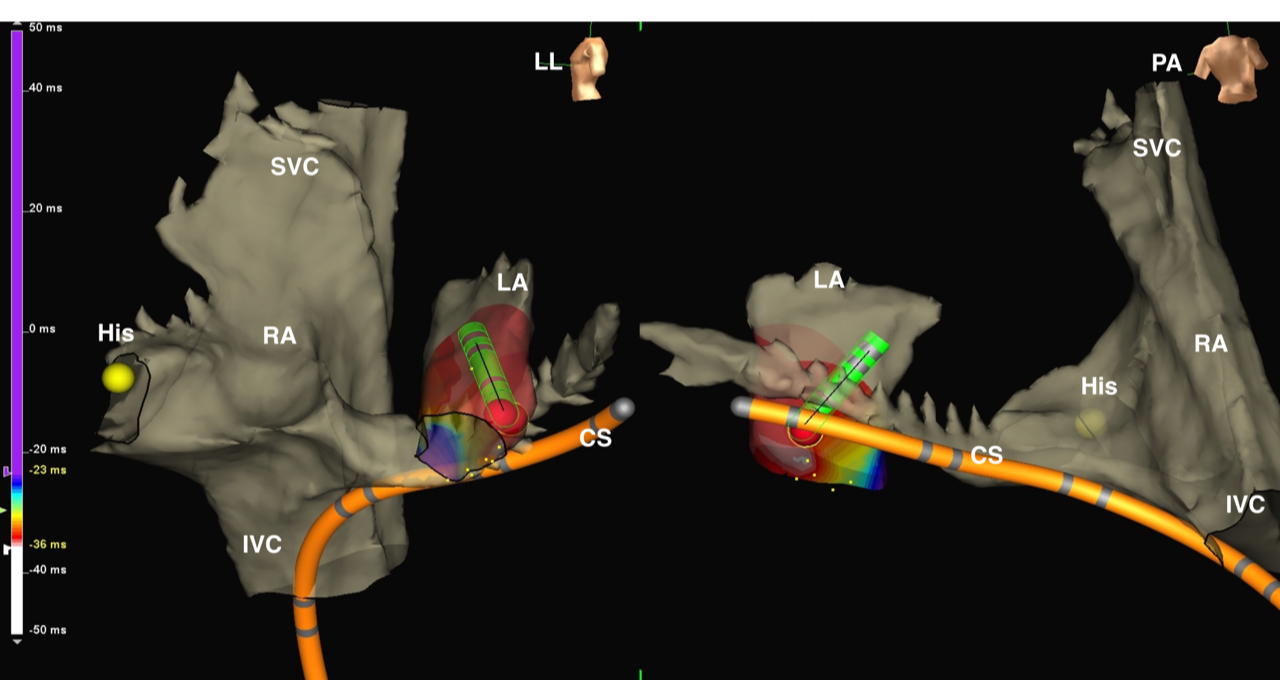
**

Figure S2: Partial anatomical 3D map of the right and left atrium with colour-coded activation sequence in the left atrium in a patient with left posterior accessory pathway. The left panel displays the left lateral view and the right panel displays the posteroanterior view. The white area marks the earliest activation in the left atrium during ongoing orthodromic atrioventricular reentry tachycardia. Red dots in the white area mark the successful radiofrequency ablation site. Also, a shadow of the ablation catheter tip is shown at the successful ablation site. CS – coronary sinus; His – His bundle; IVC – inferior vena cava; LA – left atrium; LL – left lateral; PA – posteroanterior; RA – right atrium; SVC – superior vena cava.

**
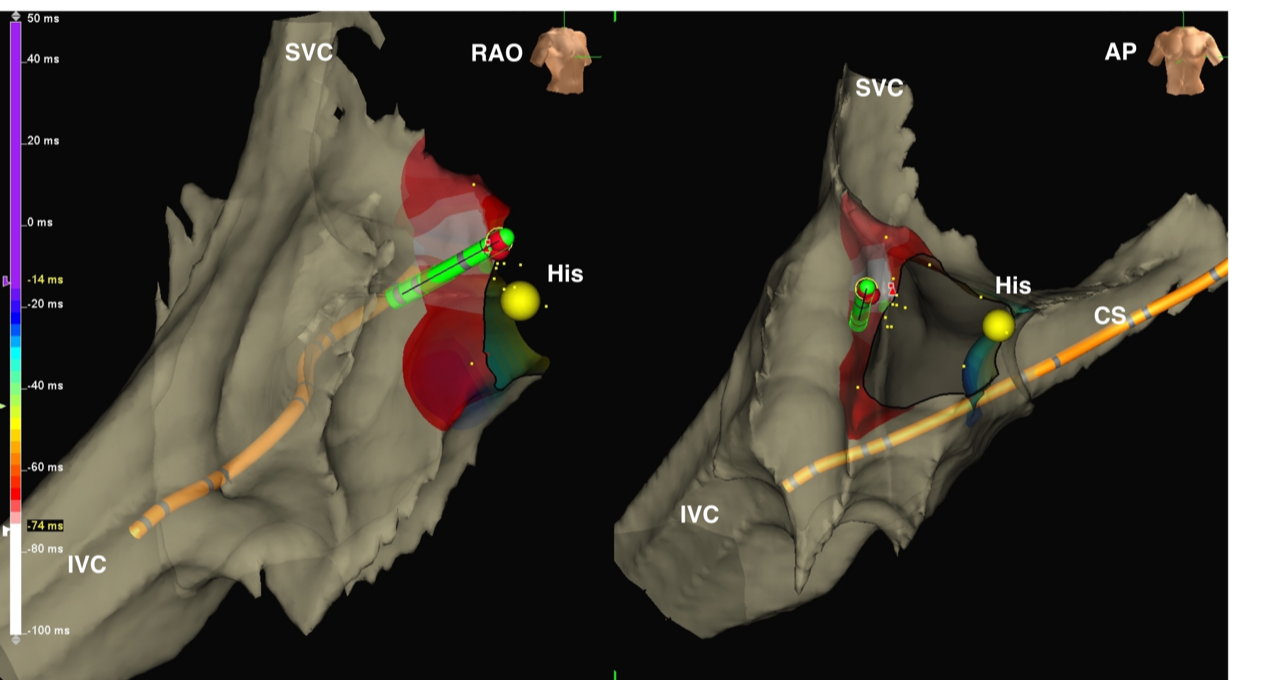
**

Figure S3: Partial anatomical 3D map of the right atrium with colour-coded activation sequence in a patient with right lateral accessory pathway. The left panel displays the right-anterior oblique view and the right panel displays the anteroposterior view. The white area marks the earliest ventricular activation during sinus rhythm at the lateral aspect of the tricuspid annulus. Red dots in the white area mark the successful radiofrequency ablation site. Also, a shadow of the ablation catheter tip is shown at the successful ablation site. AP – anteroposterior; CS coronary sinus; His – His bundle; IVC – inferior vena cava; RAO – right-anterior oblique; SVC – superior vena cava.

**
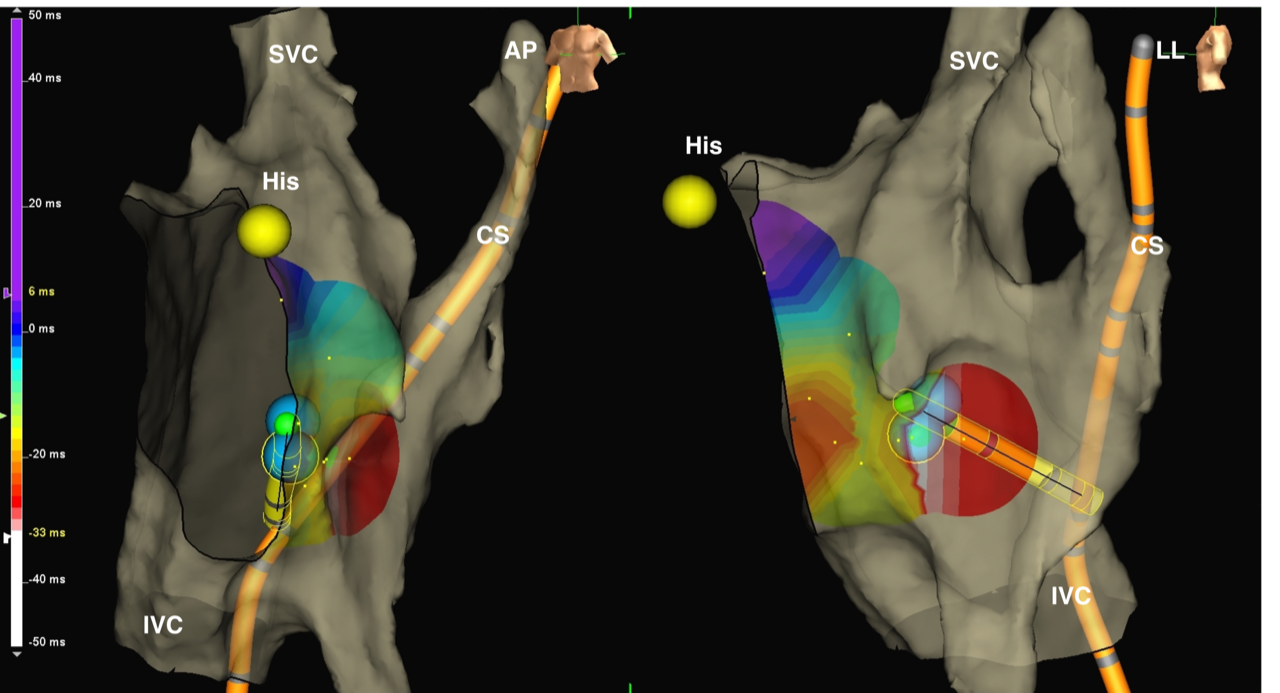
**

Figure S4: partial anatomical 3D map of the right atrium with colour-coded activation sequence in a patient with midseptal accessory pathway that was treated with cryoablation. The left panel displays the antero-posterior view and the right panel displays the left lateral view. The white area marks the earliest ventricular activation during sinus rhythm in the midseptal region. Blue dots in the white area mark the successful cryoablation site. Also, a shadow of the cryoablation catheter tip is shown at the successful ablation site. AP – anteroposterior; CS – coronary sinus; His – His bundle; IVC inferior vena cava; LL – left lateral; SVC – superior vena cava.

**
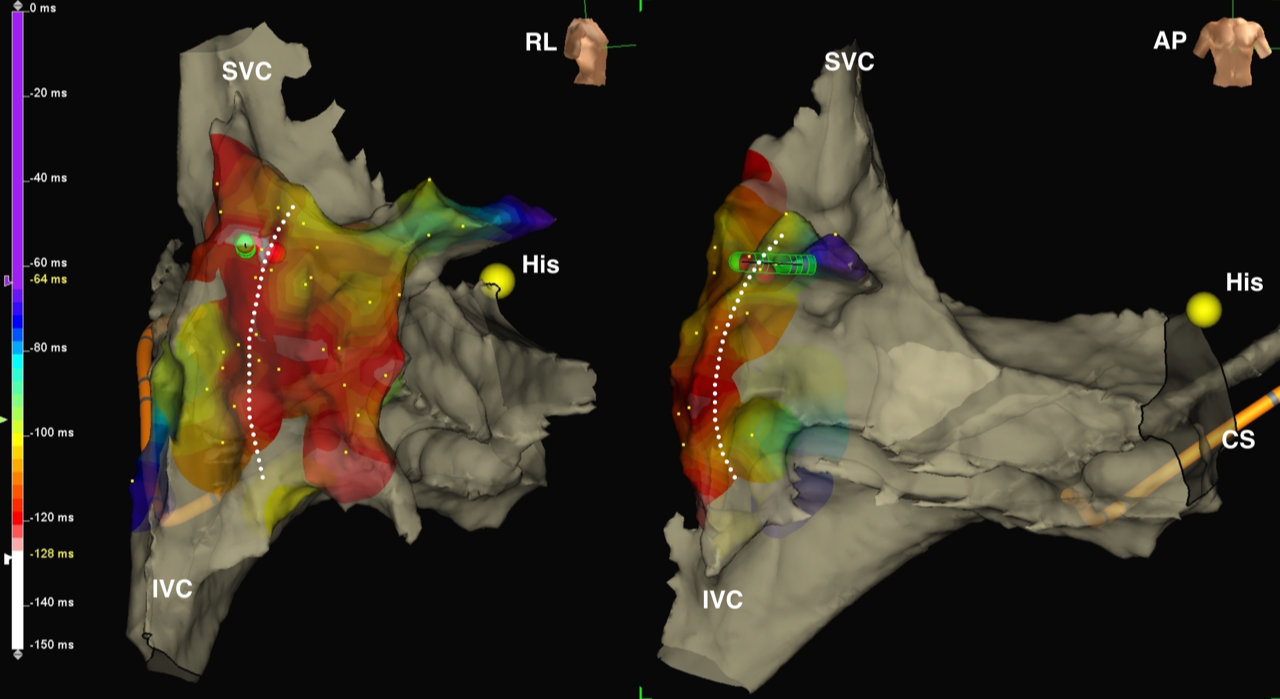
**

Figure S5: Partial anatomical 3D map of the right atrium with colour-coded activation sequence in a patient with focal atrial tachycardia originating from the upper part of the crista terminalis. The left panel displays the right lateral view and the right panel displays the anteroposterior view. The white area marks the earliest atrial activation. The dotted white line marks the course of the crista terminalis. Red dots in the white area mark the successful radiofrequency ablation site. Also, a shadow of the ablation catheter tip is shown at the successful ablation site. AP – anteroposterior; CS – coronary sinus; His – His bundle; IVC – inferior vena cava; RL – right lateral; SVC – superior vena cava.
